# Two draft genome assemblies of *Staphylococcus aureus* strains isolated from a cheek swab of a healthy female participant

**DOI:** 10.1128/mra.00488-24

**Published:** 2024-08-20

**Authors:** Alex Kula, Sandra Jablonska, Lexi Avalos, Tyler Jensen, Helen Appleberry, Catherine Putonti

**Affiliations:** 1 Department of Biology, Loyola University Chicago, Chicago, Illinois, USA; 2 Bioinformatics Program, Loyola University Chicago, Chicago, Illinois, USA; University of Maryland School of Medicine, Baltimore, Maryland, USA

**Keywords:** *Staphylococcus aureus*, cheek, oral microbiome

## Abstract

*Staphylococcus aureus* is an opportunistic pathogen often commensal within the nasal and oral cavities. Here we present the genomes of *S. aureus* O139-S and O139-NS, both isolated from the cheek swab of a healthy female participant. While found in the same sample, the two strains displayed distinct colony morphologies.

## ANNOUNCEMENT

In many healthy people, *Staphylococcus aureus* resides in the nasal and oral flora (1, 2). *S. aureus* produces a variety of immune evasion molecules and host-specific mobile genetic elements that have contributed to its persistence in different host species, not just humans [see reference (3)]. Multi-drug resistance (MDR) among *S. aureus* strains has been of concern for decades now (https://www.cdc.gov/infectioncontrol/guidelines/mdro/), and methicillin-resistant *S. aureus* (MRSA) remains a global health concern [see reference (4)]. Antibiotic resistance and even MDR rates have been identified among these “commensal” *S. aureus* strains (2, 5). Here, we present two *S. aureus* draft genome assemblies, both isolated from the cheek swab of a healthy female participant. Plating of these two strains consistently displayed different colony morphologies.

The strains were isolated from storage solution (Amies media) from an oral swab (BD BBL CultureSwab) as part of an IRB-approved study (Loyola University Chicago, #3603). Females were enrolled in the study if they were between the ages of 18 and 25 and had not taken antibiotics in the past 6 months. Samples were processed within 1 hour of receipt. Swabs were swirled and squeezed in the storage media prior to spreading the solution on Mannitol Salt Phenol Red (MS) Agar and incubated for 24 hours at 35°C with 5% CO_2_. Each distinct colony morphology was then picked and grown in MS broth and incubated with the same culture conditions. This process was repeated to purify the strain. DNA was extracted from the liquid culture using the DNeasy Blood and Tissue Kit (Qiagen), following the protocol for Gram-positive species. DNA was then sent to SeqCoast (Portsmouth, NH) where DNA libraries were constructed using the Illumina DNA Prep tagmentation kit with unique dual indexes. Libraries were sequenced on the Illumina NextSeq2000 platform producing 2 × 150 bp paired-end reads. The Bacterial and Viral Bioinformatics Resource Center (BV-BRC) webtool v3.35.5 was used to assemble the genomes with the “auto” option (6). Trim Galore v0.6.5 (https://github.com/FelixKrueger/TrimGalore) and Unicycler v0.4.8 (7) were used for trimming and assembly, respectively. Assemblies were then refined using Pilon v1.23 (8). BV-BRC was also used to calculate genome coverage, completeness, and contamination as well as conduct variant analysis. Genome annotations were produced using the NCBI Prokaryotic Genome Annotation Pipeline (PGAP) v6.7 (9). Antibiotic resistance genes were identified using ResFinder v4.5.0 (10, 11), specifying the *Klebsiella* species. Unless otherwise specified, default parameters were used for all software tools.

Table 1 lists the assembly statistics for the two strains. Variant analysis identified 25 sites of variation, including nonsynonymous mutations in annotated transposases (IS4 family) and in superantigen-encoding pathogenicity islands (SaPI). We can only speculate that these mutations contributed to the observed different colony morphologies. Both genomes encode for *blaZ*, suggestive of resistance to penicillin. The fact that the individual from whom these strains were isolated had not taken antibiotics recently may contribute to the fact that only *blaZ* was encoded in these genomes.

**TABLE 1:** Genome statistics for *S. aureus* O139-S and O139-NS

Strain	O139-S	O139-NS
No. of Raw Reads	3,037,190	2,357,462
Assembly Length (bp)	2,830,754	2,831,334
G + C (%)	32.74	32.74
No. of Contigs	69	68
Contigs N50 (bp)	150,179	150,179
Coverage (x)	139.17	117.42
Completeness (%)	100	100
Contamination (%)	0	0

## Data Availability

Raw reads are publicly available in the SRA database, with accession nos. SRR28706146 (O139-S) and SRR28706145 (O139-NS). This Whole Genome Shotgun project has been deposited in DDBJ/ENA/GenBank under the accession nos. JBCGEA000000000 (O139-S) and JBCGDZ000000000 (O138-NS). The versions described in this paper are the first version.
